# Severe Nondiabetic Hypoglycemia After Risperidone Initiation in an Adult With Schizophrenia: A Case Report and Systematic Review

**DOI:** 10.7759/cureus.106442

**Published:** 2026-04-04

**Authors:** Aditi Sarker, Muhammad Yusuf, Michael Gillespie

**Affiliations:** 1 Psychiatry, Baptist Hospitals of Southeast Texas, Beaumont, USA

**Keywords:** antipsychotic-induced hypoglycemia, case report, non-diabetic hypoglycemia, risperidone, schizophrenia, second-generation antipsychotics

## Abstract

Hypoglycemia is a rare but potentially serious adverse effect of second-generation antipsychotics (SGAs). Most studies have focused on SGA-induced hyperglycemia and metabolic syndrome, while hypoglycemia in non-diabetic adults remains underreported. We describe a case of severe hypoglycemia following risperidone initiation, demonstrating diagnostic challenges and safety concerns. A 41-year-old woman with treatment-resistant schizophrenia, Asperger’s syndrome, and attention-deficit/hyperactivity disorder was admitted for worsening commanding auditory hallucinations and persecutory delusions. Risperidone was started at 2 mg nightly and increased to 3 mg after four days. The patient developed recurrent episodes of severe hypoglycemia (random blood glucose levels 35-50 mg/dL; hypoglycemia defined as <70 mg/dL), accompanied by hypotension, tachycardia, nausea, vomiting, dizziness, and altered mental status, shortly after increasing the dose of risperidone. Extensive laboratory and imaging evaluations were unremarkable except for persistent hypoglycemia. The episodes continued despite aggressive glucose replacement and supportive medical treatment. After risperidone was discontinued, blood glucose levels gradually normalized within 48-72 hours, and no further hypoglycemic episodes occurred.

To contextualize this case, a systematic literature search was conducted following PRISMA (Preferred Reporting Items for Systematic Reviews and Meta-Analyses) guidelines using PubMed, PubMed Central, PsycINFO, Embase, and Google Scholar, with keywords including “risperidone”, “second-generation antipsychotics”, and “hypoglycemia”, as well as MeSH terms. Studies involving adult non-diabetic psychiatric patients with antipsychotic-associated hypoglycemia were included. Nine relevant case reports and case series were identified and evaluated using the Joanna Briggs Institute (JBI) Critical Appraisal Checklist for Case Reports. Existing literature shows that hypoglycemia associated with SGAs is rare but has been reported with several agents, including risperidone, quetiapine, olanzapine, paliperidone, clozapine, and aripiprazole. Proposed mechanisms include hyperinsulinemia and impaired counter-regulatory responses related to antipsychotic receptor effects. Because symptoms such as confusion, dizziness, and weakness may mimic psychiatric symptoms or medication side effects, diagnosis may be delayed. This case highlights that risperidone can rarely cause severe hypoglycemia even in non-diabetic adults. Although limited by a single case and lack of insulin measurements, this study emphasizes the need for early recognition, glucose monitoring during SGA use, and multidisciplinary care to prevent serious complications. Further research is needed to better understand the underlying biological mechanisms and identify potential risk factors.

## Introduction

Schizophrenia is a chronic mental health disability characterized by positive (hallucinations, delusions, and disorganized speech or behavior), negative (flat affect, poverty of speech, or social withdrawal), and cognitive symptoms (issues in attention, memory, or executive functioning), according to Diagnostic and Statistical Manual of Mental Disorders, 5th Edition (DSM-5) criteria [1]. Treatment-resistant schizophrenia is commonly defined as an inadequate response to at least two trials of antipsychotic medications given at an adequate dose and duration [2].

Antipsychotics are US Food and Drug Administration (FDA)-approved medications and are the mainstay treatment for schizophrenia and related disorders [3,4]. Although antipsychotics were previously divided into first-generation (typical) and second-generation (atypical) agents, the current classification is based on their mechanism of action, according to the Neuroscience-based Nomenclature (NbN) [3,4].

Second-generation antipsychotics (SGAs) are defined by their mechanism of action, primarily involving dopamine D2 receptor antagonism and serotonin 5-HT2A receptor antagonism, distinguishing them from first-generation antipsychotics, which predominantly act through dopamine D2 blockade and have a higher risk of extrapyramidal side effects [3,4].

Common SGAs include risperidone, paliperidone, olanzapine, quetiapine, and cariprazine [3]. Risperidone is a commonly prescribed SGA used for schizophrenia, bipolar disorder, and irritability associated with autism [3,4]. It acts primarily through antagonism at dopamine D2 and serotonin 5-HT2A receptors, with additional antagonistic effects at alpha-1 and alpha-2 adrenergic receptors and histamine H1 receptors [5,6]. Alpha-1 receptor blockade may contribute to hypotension, which can overlap with symptoms seen in hypoglycemia [5,6]. Risperidone is also associated with prolactin elevation due to dopamine blockade in the tuberoinfundibular pathway [5,6]. The metabolic impact of this receptor profile is not fully understood [5,6].

Compared with first-generation antipsychotics such as haloperidol and fluphenazine, SGAs are often preferred because of a lower risk of extrapyramidal (movement-related) side effects (EPS) [3,4]. However, SGAs, especially risperidone, are commonly associated with metabolic complications, including weight gain, dyslipidemia, insulin resistance, and hyperglycemia leading to diabetes mellitus [7]. Among these metabolic effects, hyperglycemia and diabetes mellitus are well recognized and frequently monitored [7]. In contrast, hypoglycemia is rarely discussed in clinical practice and is not commonly emphasized in treatment guidelines [8-16]. Hypoglycemia refers to low blood sugar, typically defined as <70 mg/dL. Severe hypoglycemia occurs when blood sugar falls below 55 mg/dL and may cause symptoms such as sweating, dizziness, confusion, and tachycardia [17]. Hypoglycemia is unexpected in non-diabetic psychiatric patients and may be misattributed to sedation, dehydration, anxiety, or worsening psychosis [8-16].

Research gap

While significant research has focused on antipsychotic-induced hyperglycemia and metabolic syndrome, there is very limited research on hypoglycemia related to risperidone use, particularly in non-diabetic adult patients. Furthermore, there are insufficient data regarding the biological mechanisms underlying this reaction, patient-related risk factors, the role of dose titration, and appropriate monitoring strategies. Because severe hypoglycemia can be life-threatening if unrecognized, increasing awareness among psychiatric providers is essential to prevent serious complications and improve patient safety.

Objectives

This study aims to describe a complex case of risperidone-induced hypoglycemia in a patient with treatment-resistant schizophrenia, highlighting diagnostic challenges and reviewing existing literature.

## Case presentation

Patient information

A 41-year-old woman with a long history of schizophrenia, a neurodevelopmental disorder (Asperger’s syndrome), and attention deficit hyperactivity disorder (ADHD) presented to the emergency department (ED) with worsening commanding auditory hallucinations and persecutory delusions. She lived with her mother and had multiple prior psychiatric hospitalizations.

History of present illness

The patient reported hearing male and female voices, perceived both internally and externally, commanding her to “go to prison and die.” Auditory hallucinations began in early childhood and increased in frequency and intensity over time. She noted partial relief with distraction, such as playing with her pet cats. She reported depressed mood and anxiety related to the hallucinations but denied suicidal or homicidal thoughts at the time of admission. She also endorsed persecutory delusions, such as people being after her and trying to harm her. The patient denied any history of substance abuse.

Past medical history

Past medical history of the patient includes scoliosis, irritable bowel syndrome (IBS), and possible polycystic ovarian syndrome.

Developmental, social, and family history

The patient’s developmental history was notable for early-onset auditory phenomena in childhood, initially musical, later evolving into commanding auditory hallucinations during adolescence. She required special education services during childhood and was subsequently diagnosed with Asperger’s syndrome. The patient was unmarried and had no children. She resided with her mother, brother, and sister in a shared household. Her family history was significant for schizophrenia in a maternal uncle, as well as cerebral palsy and autism spectrum disorder in her younger brother. She completed high school and previously volunteered at a library for approximately four years.

Mental status examination during admission

Table 1 describes the patient’s mental status examination (MSE) on admission, providing a structured assessment of her appearance, behavior, speech, mood, affect, thought processes and content, insight, and judgment to guide diagnosis and treatment. 

**Table 1:** Mental status examination on admission.

Domain	Findings
Appearance	41-year-old middle aged, Caucasian female, casually dressed, well nourished, well-groomed, no signs of neglect
Manner/Behavior	Cooperative but anxious and restless; repetitive finger movements; intermittent eye contact
Speech	Normal rate, tone, and volume; fluent
Motor Activity	Psychomotor agitation noted; no abnormal involuntary movements apart from finger movements
Mood	Subjectively mildly depressed and anxious due to hallucinations and family stress
Affect	Restricted but appropriate; sometimes matches mood
Thought Process	Largely linear, coherent, and goal-directed; follows conversation well
Thought Content	Auditory hallucinations (male and female voices commanding her to “go to prison and die”); paranoid delusions; no suicidal or homicidal thoughts at admission
Insight	Limited; partially aware hallucinations are abnormal, some attribution to external forces
Judgment	Impaired in daily functioning due to psychotic symptoms; difficulty living independently

Collateral and past psychiatric history

Collateral history from her mother revealed that the patient was diagnosed with Asperger’s syndrome at the age of six, schizophrenia at age 19, and had a history of ADHD during adolescence. She had multiple prior medication trials, including clozapine for six months (discontinued due to worsening restlessness), quetiapine for an extended period (caused constipation), haloperidol for one year (with limited effect), and benzodiazepine (diazepam, with a side effect of drooling). The patient’s current home medications are cariprazine 6 mg daily, asenapine 10 mg BID (two antipsychotics due to worsening psychosis), lithium ER 300 mg BID (mood stabilizer), clorazepate 7.5 mg four times a day (a long-acting benzodiazepine), and atomoxetine 60 mg daily (a non-stimulant used for ADHD). She had been on these medications for five years to stabilize her mood, restlessness, anxiety, and psychosis. The patient was admitted to another behavioral hospital in 2020 due to worsening psychosis and received approximately 30 sessions of electroconvulsive therapy (ECT) with limited benefit. She also had two prior episodes of catatonia (lasting one to two weeks) and brief mood swings lasting a few hours, without sustained depressive or manic episodes. Her history did not meet criteria for major depressive disorder, mania or hypomania, anxiety disorders, or post-traumatic stress disorder. The mother denied any history of substance abuse.

Psychiatric home medication list

The medication list included cariprazine 6 mg daily, asenapine 10 mg BID, lithium ER 300 mg BID, clorazepate 7.5 mg four times a day, and atomoxetine 60 mg daily.

Hospital course and medication changes

The patient was admitted to the acute inpatient psychiatric unit. On admission, she was anxious but cooperative, with a linear thought process. Initial physical and neurological examinations were unremarkable, and vital signs were stable. Her initial blood glucose level was 83 mg/dL. She reported commanding auditory hallucinations but denied suicidal intent. Risperidone 2 mg nightly was started during admission and increased to 3 mg nightly after four days. Cariprazine 6 mg once daily and atomoxetine 60 mg once daily were continued. Lithium and asenapine were discontinued. Clorazepate was replaced with clonazepam 0.5 mg three times a day, which was gradually titrated. During the first four to five days, psychotic symptoms improved.

Acute medical deterioration

Following a stressful family meeting, the patient developed severe hypotension (BP 85/58 mmHg), tachycardia (HR 114 bpm), nausea, vomiting, diarrhea, and recurrent hypoglycemia (random blood glucose 35-50 mg/dL), accompanied by altered mental status characterized by confusion, lethargy, and decreased responsiveness. These symptoms emerged shortly after risperidone dose escalation (within 12-24 hours) and were most notable during the overnight and early morning periods. All glucose measurements were obtained as random values using point-of-care testing.

Management included repeated administration of 40% oral glucose gel (one tube before meals and at bedtime) and frequent finger-stick glucose monitoring. Due to persistent hypotension, the patient received a one-time 1000 mL intravenous normal saline bolus under close supervision. Additional supportive care consisted of dietary modifications, including intermittent snacks and two servings of sugary juice daily to help prevent further hypoglycemic episodes. An internal medicine consultation was obtained, and a 10-day course of oral prednisone (10 mg daily) was initiated; however, hypoglycemia persisted despite these interventions.

Medical workup

To evaluate the patient’s acute hypoglycemia and associated clinical symptoms, a series of laboratory investigations was performed, including metabolic panels, pancreatic enzymes, and imaging studies. These results, summarized in Table 2, helped rule out other potential causes of hypoglycemia and guided clinical management.

**Table 2:** Laboratory investigations during hospital admission highlighting recurrent hypoglycemia and associated findings. CK: creatinine kinase, ESR: erythrocyte sedimentation rate, and CRP: C-reactive protein.

Investigation	Result	Reference Range	Comments
Complete blood count (CBC)	Normal	Varies by lab	No hematologic abnormalities
Comprehensive metabolic panel (CMP)	Normal	Varies by lab	Liver and kidney functions within normal limits
Random blood glucose	35–50 mg/dL (during hypoglycemia)	70–140 mg/dL (random)	Recurrent hypoglycemia episodes, mostly after risperidone at bedtime
Amylase	Normal	30–110 U/L	No pancreatic abnormality
Lipase	Normal	0–160 U/L	No pancreatic abnormality
CT abdomen	Normal	NA	No structural abnormality
Abdominal ultrasound	Fatty liver	NA	Incidental finding, otherwise normal
Other labs (CK, inflammatory markers such as ESR and CRP)	Normal	CK (78), ESR (0-20 mm/h in females), and CRP (<0.3 mg/dL)	No infection identified

Medication adjustment and outcome

Despite aggressive medical management, including glucose protocols (oral 40% glucose gel, one tube before meals and at bedtime), dietary modifications (intermittent snacks and two servings of sugary juice daily), intravenous fluids (one-time 1000 mL normal saline bolus), prednisone (10 mg daily for 10 days), and internal medicine consultation, her hypoglycemia persisted for 14 days. During this period, cariprazine was initially reduced to 2 mg daily to help stabilize blood pressure, but psychotic symptoms, including hallucinations and internal preoccupation, became more prominent. Hypoglycemia developed within approximately 12-24 hours following nighttime dose escalation and was most pronounced during overnight and early morning hours. After clinical consideration and literature review, risperidone was discontinued. Following discontinuation, the patient’s blood glucose stabilized (up to 108 mg/dL at bedtime), and associated symptoms, including hypotension, tachycardia, nausea, vomiting, and altered mental status, resolved. No further hypoglycemic episodes occurred, and the patient’s diet returned to normal. At discharge, her blood glucose level was 110 mg/dL.

To manage her psychosis, cariprazine was reintroduced at 6 mg daily, and olanzapine 5 mg at bedtime was added initially. Olanzapine was later titrated to 15 mg at bedtime, while cariprazine was cross-titrated and subsequently discontinued. The patient’s mood and psychosis were ultimately stabilized with olanzapine 15 mg at bedtime. Clonazepam and atomoxetine were gradually tapered and discontinued before discharge. The patient was maintained on a single antipsychotic (olanzapine 15 mg at bedtime) to reduce polypharmacy, and her mood and psychotic symptoms stabilized. Table 3 summarizes the patient’s hospital course, including the initiation of risperidone, the onset and resolution of hypoglycemia, subsequent medication adjustments, the transition to olanzapine monotherapy with discontinuation of prior home medications, and follow-up outcomes. This timeline provides a clear overview of interventions and responses throughout her admission.

**Table 3:** Timeline of hospital course highlighting risperidone initiation, dose escalation, onset of hypoglycemia and associated clinical symptoms, and subsequent clinical improvement following medication adjustment and discontinuation.

Day	Clinical Events/Medication Changes	Notes/Observations
Day 1	Admission to acute psychiatric unit; baseline random blood glucose 83 mg/dL; Risperidone 2 mg nightly started.	Patient anxious but cooperative; linear thought process; psychotic symptoms present
Day 4	Risperidone dose increased to 3 mg nightly	No immediate adverse effects noted
Days 5–7	Onset of recurrent hypoglycemia (random blood glucose 35–50 mg/dL), hypotension (BP 85/58 mmHg), tachycardia (HR 114 bpm), nausea, vomiting, dizziness, and altered mental status (confusion, lethargy)	Followed hypoglycemia protocol in the hospital; glucose gel (40%) administered before meals and at bedtime; dietary modifications (snacks + 2 sugary juices/day); one-time IV NS 1000 mL; prednisone 10 mg daily started; internal medicine consulted.
Days 1–10	Prednisone 10 mg daily prescribed by internal medicine (discontinued after 10 days)	Prednisone was discontinued due to minimal benefit.
Days 8–14	Continued hypoglycemia despite interventions; Cariprazine was temporarily reduced to 2 mg daily to stabilize BP.	It was found that hypoglycemia predominantly occurred after bedtime with Risperidone. Psychosis was more prominent after Cariprazine reduction.
Day 14	Risperidone discontinued	Blood glucose started to improve within 48–72 hours (up to 108 mg/dL at bedtime); hypotension, tachycardia, nausea, vomiting, and altered mental status resolved.
Days 15–20	Cariprazine reintroduced at 6 mg daily; Olanzapine 5 mg at bedtime added	Initial management of psychosis; cross-titration plan implemented
Days 21–36 and discharge	Olanzapine titrated to 15 mg at bedtime; Cariprazine cross-titrated and discontinued; Clonazepam and Atomoxetine tapered and discontinued	Patient maintained on single antipsychotic (Olanzapine 15 mg nightly); mood and psychotic symptoms stabilized. During discharge, her blood glucose level was 110 mg/dL.
Follow-up (2 weeks)	Follow-up in 2 weeks	Patient reported to be doing well with no recurrence of mental or physical symptoms.

**Figure 1:**
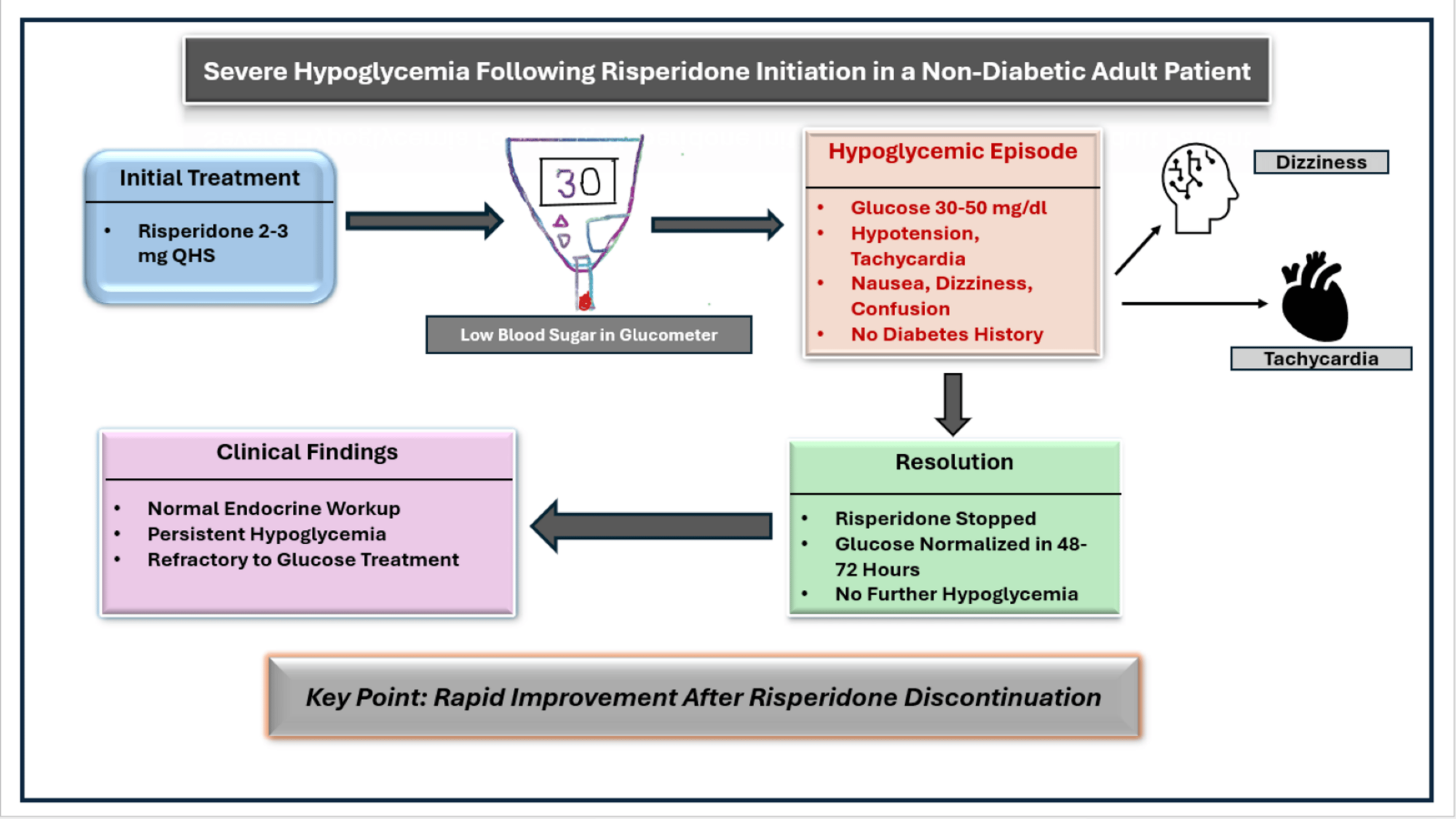
Clinical timeline showing risperidone initiation and the development of recurrent hypoglycemia in the patient. The sequence of medication initiation, onset of symptoms, and clinical management is illustrated. Image created by the primary author using Microsoft PowerPoint (Microsoft Corporation, Redmond, Washington).

The clinical course and timeline of risperidone initiation and the development of hypoglycemia in this patient are summarized in Figure 1. Image created by the primary author only using Microsoft PowerPoint.

## Discussion

Informed written consent and search strategy

Written informed consent was obtained from the patient before the study. The research question for this case report was to examine the temporal association between risperidone initiation and the development of severe hypoglycemia in a non-diabetic adult patient. To address this, a systematic literature search was performed to identify previously reported cases of hypoglycemia associated with second-generation antipsychotics (SGAs), with a particular focus on risperidone. We followed the Preferred Reporting Items for Systematic Reviews and Meta-Analyses (PRISMA) guidelines [18].

Electronic databases, including PubMed, PsycINFO, Embase, and Google Scholar, were searched using keywords such as “second-generation antipsychotics,” “risperidone,” “hypoglycemia,” “insulin resistance,” “schizophrenia,” and “metabolic syndrome.” Additionally, Medical Subject Headings (MeSH) terms were used to obtain more precise search results:

Risperidone OR second-generation antipsychotic OR (“Risperidone/adverse effects” [Majr] OR “Risperidone/pharmacokinetics” [Majr] OR “Risperidone/pharmacology” [Majr] OR “Risperidone/therapeutic use” [Majr] OR “Risperidone/toxicity” [Majr]) AND (“Antipsychotic Agents/adverse effects” [Majr] OR “Antipsychotic Agents/pharmacokinetics” [Majr] OR “Antipsychotic Agents/pharmacology” [Majr] OR “Antipsychotic Agents/therapeutic use” [Majr] OR “Antipsychotic Agents/toxicity” [Majr]) AND (“Hypoglycemia/diagnosis” [Majr] OR low blood glucose OR “Hypoglycemia/drug therapy” [Majr] OR “Hypoglycemia/etiology” [Majr] OR “Hypoglycemia/metabolism” [Majr] OR “Hypoglycemia/physiopathology” [Majr]) AND (“Schizophrenia Spectrum and Other Psychotic Disorders/complications” [Majr] OR “Schizophrenia Spectrum and Other Psychotic Disorders/drug therapy” [Majr] OR “Schizophrenia Spectrum and Other Psychotic Disorders/etiology” [Majr] OR “Schizophrenia Spectrum and Other Psychotic Disorders/physiopathology” [Majr]).

Two reviewers (AS and MY) independently screened the titles and abstracts of each article to determine eligibility. They then compared their decisions and resolved any disagreements through discussion.

Inclusion and exclusion criteria

For this review, we included studies that reported adult patients (aged 18-80 years) with primary psychotic disorders who developed hypoglycemia after receiving SGAs such as risperidone, olanzapine, quetiapine, aripiprazole, or paliperidone. Only patients without diabetes or other endocrine disorders were included. Studies were required to report blood glucose measurements or clinical symptoms of hypoglycemia. We included only case reports and case series published in peer-reviewed journals between 2006 and 2026 and written in English.

We excluded studies involving children, patients with diabetes, patients without a psychiatric disorder, and those receiving other medications known to affect blood glucose levels.

Risk of bias assessment of the studies

Two investigators (AS and MY) independently assessed the risk of bias using the Joanna Briggs Institute (JBI) Critical Appraisal Checklist for Case Reports [19]. Table 4 summarizes the results of the quality assessment. The quality appraisal score for each study was calculated as the percentage of “Yes” responses out of the total eight questions in the checklist. Scores ranged from 87.5% to 100%, indicating a low risk of bias across the included studies.

**Table 4:** Quality assessment of case reports using JBI critical appraisal checklist.

Study	Type of Article	Tool Used	No. of Questions	Yes Responses	Risk of Bias Assessment	Study Quality
Fujita et al., 2018 [8]	Case Report	JBI Case Report Checklist	8	8	Low	High
Nagamine, 2016 [9]	Case Report	JBI Case Report Checklist	8	8	Low	High
Omi et al., 2016 [10]	Case Report	JBI Case Report Checklist	8	8	Low	High
Ochi et al., 2020 [11]	Case Report	JBI Case Report Checklist	8	8	Low	High
Suzuki et al., 2009 [12]	Case Report	JBI Case Report Checklist	8	8	Low	High
Mondal et al., 2012 [13]	Case Report	JBI Case Report Checklist	8	7	Low	High
Kawai et al., 2023 [14]	Case Report	JBI Case Report Checklist	8	8	Low	High
Nagamine, 2006 [15]	Case Report	JBI Case Report Checklist	8	8	Low	High
Ishiguro et al., 2015 [16]	Case Report	JBI Case Report Checklist	8	8	Low	High

Literature search and study selection

The initial database search identified 316 articles: 26 from PubMed, 35 from Embase, 22 from PsycINFO, and 233 from Google Scholar. After removing 218 duplicate records, 98 unique articles remained for title and abstract screening. Of these, 52 records were excluded because they did not meet the inclusion criteria, including studies on children, patients with diabetes or other endocrine disorders, non-psychiatric populations, or studies not reporting on second-generation antipsychotics. An additional 27 records could not be retrieved in full text.

The remaining 19 full-text articles were assessed for eligibility. Of these, 10 articles were excluded because they either did not report hypoglycemia associated with second-generation antipsychotics or lacked sufficient clinical details. The final review included nine articles, from which key information was extracted, including patient demographics, antipsychotic type and dose, onset and severity of hypoglycemia, laboratory results, and management or outcomes.

**Figure 2:**
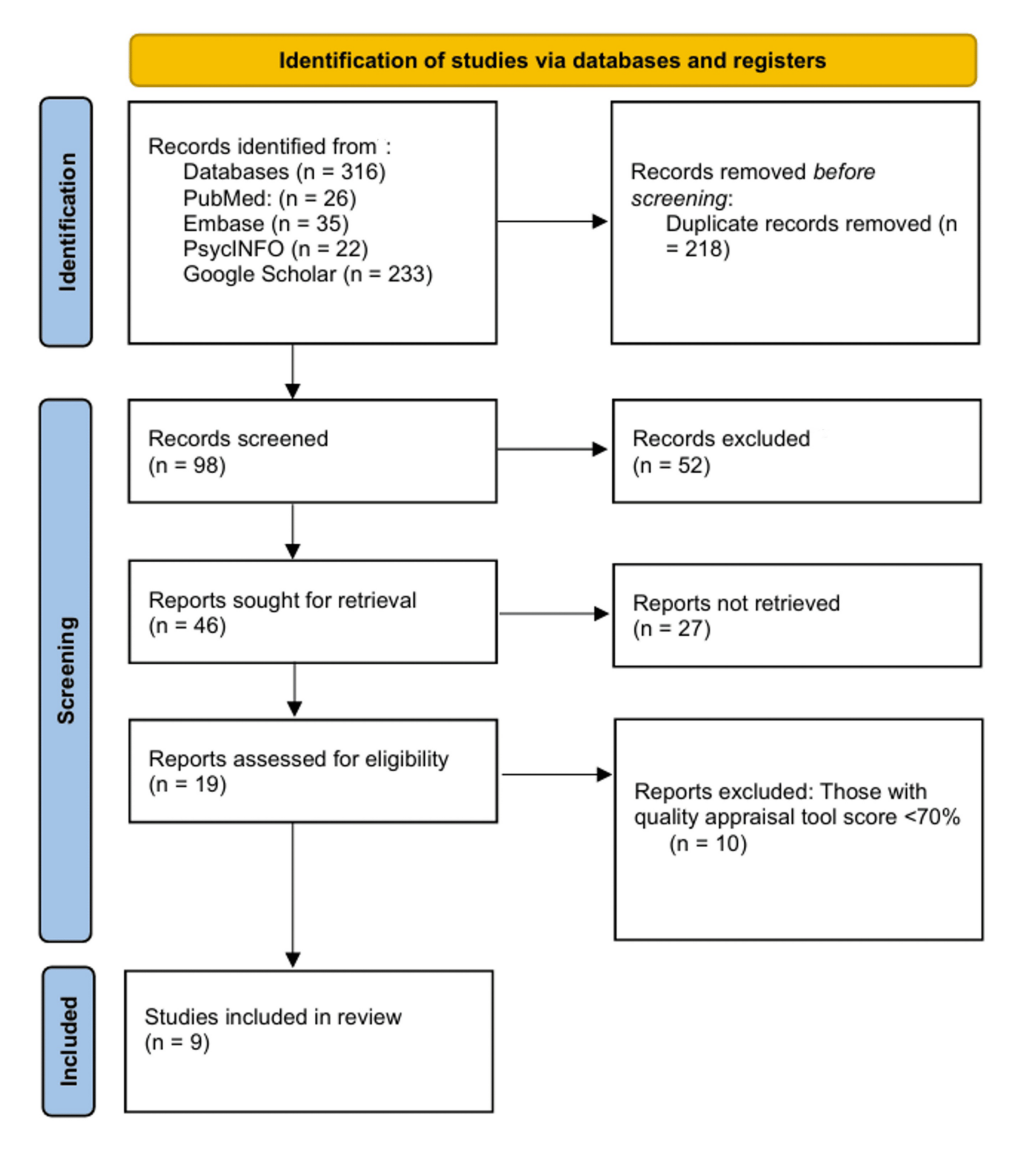
PRISMA flow diagram demonstrating the process of article selection. PRISMA: Preferred Reporting Items for Systematic Reviews and Meta-Analyses.

Quality appraisal scores for the included studies were calculated as the percentage of “Yes” responses on the JBI Critical Appraisal Checklist for Case Reports, ranging from 87.5% to 100%, indicating a low risk of bias across all studies. Figure 2 presents the PRISMA 2020 flow diagram for article identification, screening, and selection, with quality appraisal scores displayed alongside the included studies.

Table 5 summarizes the key characteristics of patients reported in the included studies who developed hypoglycemia after receiving second-generation (atypical) antipsychotics. Information includes study type, antipsychotic agent and dose, patient age, onset of hypoglycemia after initiation of the medication, severity of hypoglycemia as indicated by blood glucose levels, and management or outcomes. This provides an overview of the clinical presentation and treatment response across reported cases.

**Table 5:** Clinical characteristics of reported cases of hypoglycemia associated with second-generation antipsychotics.

First Author (Year)	Type of Article	Antipsychotic Agent	Patient Age (Years) and Sex	Onset of Hypoglycemia After Initiation	Severity/Glucose Level	Outcome/Management
Fujita et al., 2018 [8]	Case Report	Quetiapine 25 mg/day	62, Female	Hypoglycemia shortly after quetiapine initiation	Fasting hypoglycemia (69 mg/dL after meals)	Discontinued quetiapine; switched to risperidone; hypoglycemia resolved
Nagamine, 2016 [9]	Case Report	Risperidone 2 mg/day, Quetiapine 50 mg/day	77, Male	4 weeks after initiation	Severe early morning hypoglycemia, fasting BG 24–42 mg/dL	Switched to Blonanserin; hypoglycemia resolved
Omi et al., 2016 [10]	Case Report	Paliperidone 12 mg/day	41, Female	Persistent hypoglycemia during paliperidone use	Fasting plasma glucose 51 mg/dL with elevated insulin	Reduced/discontinued paliperidone; glucose improved
Ochi et al., 2020 [11]	Case Report	Risperidone 6 mg/day & Olanzapine 10 mg/day	60, Male	2 weeks after starting risperidone; next day after olanzapine	Hypoglycemic coma (Blood Glucose 50 mg/dL) on both agents	Changed to haloperidol; hypoglycemia resolved
Suzuki et al., 2009 [12]	Case Series	Quetiapine 600 mg/day, Risperidone, Olanzapine 20 mg/day	27, Female;32, Female and 53, Male (series)	During dose escalation	Post‑meal hypoglycemia (45-55 mg/dl)	Dose reduction or switch to Perospirone/other; symptoms improved
Mondal et al., 2012 [13]	Case Report	Aripiprazole (NA)	72, Male	10 days after aripiprazole initiation; worsened by day 21	Severe hypoglycemia requiring hospitalization	Dechallenge/rechallenge confirmed association; stopping aripiprazole resolved events
Kawai et al., 2023 [14]	Case Report	Clozapine 500 mg/dl	50, Male	8 months after dose escalation to 500 mg	Reactive hypoglycemia on OGTT (postprandial drop to 40–50 mg/dL)	Voglibose & diet therapy; clozapine continued with management
Nagamine et al., 2006 [15]	Case Report	Olanzapine 10-15 mg/day	47, Male	4 days after adding olanzapine	Multiple early‑morning hypoglycemic episodes (plasma glucose 44–57 mg/dL)	Discontinued olanzapine; no further episodes
Ishiguro et al., 2015 [16]	Case Report	Olanzapine, Quetiapine, Paliperidone	55, Male	Recurrent episodes while on multiple atypical antipsychotic drugs (APD)	Symptomatic hypoglycemia with low glucose	Stopped atypical APDs; hypoglycemia ceased

Overview of findings

This case of severe, persistent hypoglycemia in a non-diabetic adult occurred shortly after initiation and titration of risperidone. It resolved only after the medication was discontinued, strongly suggesting risperidone-induced hypoglycemia. Although SGAs are widely associated with metabolic effects such as weight gain, hyperglycemia, and incident diabetes, especially compared with first-generation antipsychotics, hypoglycemia is far less recognized and may be underreported due to diagnostic challenges [4,7,8-16].

Metabolic effects of second-generation antipsychotics (SGAs)

SGAs are widely known to cause weight gain, hyperglycemia, and diabetes [7]. However, hypoglycemia is rarely discussed and may be underrecognized as a life-threatening complication of SGAs [8-16]. Our case adds to the growing evidence that risperidone can cause severe hypoglycemia even in non-diabetic adult patients.

SGAs have a well-established risk for metabolic dysregulation, including increased risks of hyperglycemia and diabetes compared with first-generation antipsychotics, which are less frequently associated with metabolic adverse events but more associated with extrapyramidal symptoms [4,7]. SGAs were designed to reduce neurological side effects, but they can also affect insulin levels, blood sugar control, and body fat [4,7].

Previously reported cases of SGA-induced hypoglycemia and comparison with the current case

Previous reports support the link between SGAs and hypoglycemia, typically associated with initiation or dose escalation. Quetiapine-induced hypoglycemia was reported in both Fujita et al. (2018) and Suzuki et al. (2009), presenting as fasting or postprandial hypoglycemia that resolved after dose reduction or switching to an alternative antipsychotic [8,12]. Risperidone-associated hypoglycemia is less frequently reported; however, Nagamine (2016) described a 77-year-old man who developed severe early morning hypoglycemia while receiving a combination of risperidone and quetiapine, which resolved after switching to blonanserin [9]. In contrast, several other reports have described hypoglycemia occurring with single-agent SGAs, suggesting that both monotherapy and combination therapy may contribute to this adverse effect. Similarly, our case highlights that risperidone can induce rapid-onset hypoglycemia even at low to moderate doses, emphasizing the need for vigilance.

Other SGAs, including paliperidone and olanzapine, have also been implicated. Omi et al. (2016) reported paliperidone-induced hypoglycemia with elevated insulin levels, while Nagamine et al. (2006) and Ishiguro et al. (2015) described symptomatic hypoglycemia with olanzapine [10,15,16]. Aripiprazole (Mondal et al., 2012) and clozapine (Kawai et al., 2023) have been associated with postprandial or reactive hypoglycemia, which improved after discontinuation or with dietary or pharmacologic management [13,14]. Ochi et al. (2020) reported severe hypoglycemic coma in a patient receiving both risperidone and olanzapine, which resolved after switching to haloperidol [11].

Our case is unique and particularly important because severe hypoglycemia occurred after dose escalation of risperidone, strengthening the temporal association with the medication. Notably, hypoglycemia developed within approximately 12-24 hours following nighttime dose escalation and was most pronounced during overnight and early morning hours. The hypoglycemia was particularly severe (as low as 35 mg/dL) and caused hemodynamic instability, including hypotension, tachycardia, and dizziness. Severe hypoglycemia can lead to seizures, neurological injury, coma, or death if not recognized and treated promptly [17].

The patient was not elderly, not malnourished, and had no prior diabetes, which strongly suggests a direct drug effect of risperidone. This highlights a rare but potentially life-threatening adverse effect and emphasizes the importance of clinical awareness when prescribing risperidone, especially given its widespread use in patients with schizophrenia. Although olanzapine has more frequently reported metabolic side effects, including glucose dysregulation, it was selected based on clinical judgment regarding efficacy and tolerability in treatment-resistant psychosis. Blood glucose was closely monitored due to its known metabolic effects.

Comparison with first-generation antipsychotics

While first-generation antipsychotics tend to have a lower metabolic risk profile, they exhibit higher rates of extrapyramidal side effects (e.g., acute dystonia, akathisia). In contrast, SGAs such as risperidone, quetiapine, and olanzapine are associated with increased risks of metabolic effects, including glucose dysregulation [4,5,7]. This explains why SGAs are commonly used despite the rarity of hypoglycemia; they are safer for the nervous system but require metabolic monitoring [4,7].

Proposed biological mechanisms

Antipsychotic-induced hypoglycemia is thought to occur mainly due to hyperinsulinemia. Previous research studies reported cases of severe hypoglycemia with SGAs and found that many patients had a high insulin-to-glucose ratio, suggesting excessive insulin secretion [8-16]. Supporting this, Karczewska-Kupczewska et al. (2009) showed that hyperinsulinemia can strongly suppress serum ghrelin, a hormone involved in glucose regulation, indicating that increased insulin activity can disrupt normal glucose control and contribute to hypoglycemia [20].

Antipsychotic-induced hypoglycemia is hypothesized to occur through excessive insulin secretion, possibly mediated by antagonism of alpha-2 adrenergic receptors and D2 receptors on pancreatic beta cells, which normally inhibit insulin secretion [9]. Some SGAs also antagonize muscarinic receptors, potentially promoting continued insulin secretion after glucose levels normalize [9].

These receptor interactions may alter pancreatic beta-cell function, leading to inappropriate insulin release and impaired counter-regulatory responses [9]. Even though we did not measure insulin in our patient, the timing of hypoglycemia with risperidone and its improvement after stopping the drug support this mechanism.

Diagnostic challenges in psychiatric settings

Another important issue is diagnostic difficulty. Symptoms of hypoglycemia, such as confusion, anxiety, tremors, sweating, dizziness, weakness, drowsiness, and altered mental status, can easily be mistaken for psychiatric symptoms or medication side effects [11,12,17]. As noted in previous reports, clinicians may attribute these symptoms to sedation, anxiety, or worsening psychosis.

In psychiatric inpatient settings, behavioral changes are often first interpreted as psychiatric in origin [1]. In our patient, the altered mental status and physical symptoms could have been misattributed to emotional stress or worsening psychosis if blood glucose had not been checked.

Clinical significance

Severe low blood sugar can cause autonomic symptoms (such as a fast heart rate and low blood pressure) and neuroglycopenic symptoms (such as confusion, fainting, or seizures). It can even be life-threatening if untreated [17]. Risperidone may also contribute to low blood pressure through alpha-1 receptor effects [6].

SGA-induced hypoglycemia is rare but can occur with multiple second-generation antipsychotics, including risperidone, paliperidone, quetiapine, olanzapine, aripiprazole, and clozapine [8-16]. This case highlights the importance of recognizing a rare metabolic side effect during antipsychotic treatment. It emphasizes the need for close collaboration between psychiatry and internal medicine when unexplained medical symptoms, such as hypoglycemia, develop. Awareness of this uncommon but potentially life-threatening adverse effect is essential to ensure patient safety. Future research should track side effects of antipsychotics, identify which patients are at higher risk, and explore how these drugs affect insulin and blood sugar control. Such studies could help develop better guidelines for safe glucose monitoring and reduce serious complications during antipsychotic treatment.

Limitations

This case report has several limitations. Insulin and C-peptide levels were not measured during the hypoglycemic episodes because the patient was admitted to an inpatient psychiatric unit, and frequent transfers to the ER were difficult due to weakness, fatigue, and episodes of falls caused by worsening hypoglycemia, which limits confirmation of the exact biological mechanism. A rechallenge with risperidone was not performed due to ethical and safety concerns. In addition, because this is a single case report, the findings cannot be generalized to all patients, and a definite causal relationship cannot be established. Despite these limitations, the strong temporal association and post-discontinuation resolution support a likely medication-related effect.

## Conclusions

This case shows that risperidone can rarely cause severe hypoglycemia in non-diabetic adults. The close timing between risperidone initiation, titration, and the onset of recurrent hypoglycemia, along with clear improvement after discontinuation, supports a probable drug-induced reaction. Clinicians should recognize that while hyperglycemia remains more commonly associated with SGAs, hypoglycemia can occur and may have life-threatening consequences. Monitoring blood glucose, especially during initiation or dose escalation, and maintaining interdisciplinary care are important strategies to improve patient safety. Further research on underlying mechanisms, risk stratification, and monitoring protocols is needed. Larger clinical and pharmacological studies may help guide future monitoring recommendations.
